# Longitudinal pathway analysis using structural information with case studies in early type 1 diabetes

**DOI:** 10.1038/s41598-025-98492-0

**Published:** 2025-05-02

**Authors:** Maria K. Jaakkola, Anu Kukkonen-Macchi, Tomi Suomi, Laura L. Elo

**Affiliations:** 1 Turku Bioscience Centre, University of Turku and Åbo Akademi University, Turku, Finland; 2 Department of Mathematics and Statistics, University of Turku, Turku, Finland; 3 Institute of Biomedicine, University of Turku, Turku, Finland

**Keywords:** Pathway analysis, Computational method, Longitudinal data, Gene expression, Protein expression, Type 1 diabetes, Gene regulatory networks, Software, Molecular medicine

## Abstract

Pathway analysis is a frequent step in studies involving gene or protein expression data, but most of the available pathway methods are designed for simple case versus control studies of two sample groups without further complexity. The few available methods allowing the pathway analysis of more complex study designs cannot use pathway structures or handle the situation where the variable of interest is not defined for all samples. Such scenarios are common in longitudinal studies with so long follow up time that healthy controls are required to identify the effect of normal aging apart from the effect of disease development, which is not defined for controls. To address the need, we introduce a new method for Pathway Analysis of Longitudinal data (PAL), which is suitable for complex study designs, such as longitudinal data. The main advantages of PAL are the use of pathway structures and the suitability of the approach for study settings beyond currently available tools. We demonstrate the performance of PAL with simulated data and three longitudinal datasets related to the early development of type 1 diabetes, which involve different study designs and only subtle biological signals, and include both transcriptomic and proteomic data. An R package implementing PAL is publicly available at https://github.com/elolab/PAL.

## Introduction

Pathway analysis is a routine step when analysing gene or protein expression data and many tools have been developed for this purpose. Most of these tools are very simple and provide either a list of pathways with different activity between case and control sample groups (e.g.^1–6^), or pathway scores for each sample as such (e.g.^7–12^). Control samples serve as a reference point for the normal situation without the phenomenon under study (e.g. no disease, no drug given, or no particular mutation or exposure to some environmental factor under study) and they are sometimes called reference samples in the literature. However, many research projects involving transcriptomic or proteomic data have a more complex study design, making the currently available pathway methods unsuited for them. Moreover, the majority of the available pathway methods, including all of the aforementioned examples, have been designed and validated using transcriptomic data only.

Here we introduce a novel method for Pathway Analysis of Longitudinal data (PAL), which is specifically designed for complex study designs. PAL can identify pathways with significant associations with a user-defined main outcome after adjusting for the effect of given confounding variables. Both the confounding variables and the main outcome variable can be either numeric or categorical, which supports the versatility of the method. PAL is suited to analyze longitudinal data and it can handle non-aligned time points, which is particularly important in studies involving long follow-up times, where the measured ages of different sample donors may not match. While longitudinal data is the most important application for PAL, it can also be applied to other types of study settings, including simple ones, such as multiple categorical sample groups. A major advantage of PAL is that it can estimate the significance of pathways related to outcome variables that are only defined for case samples (e.g. event time or disease stage) and still control for relevant confounding variables that are present for all samples (e.g. age in studies with long follow-up time). Thus, PAL can also utilize the control samples even if the variable of interest is not defined for them. As far as we know, no other pathway method enables this. Another major advantage of PAL is its ability to use pathway structures, which is important as methods utilizing pathway structures have been shown to outperform simple gene set approaches in the context of straightforward case versus control comparisons^13–15^. In this study, we also demonstrate that, besides transcriptomic data, PAL can be applied successfully to proteomic data, which further expands its potential applications.

There are relatively few methods that allow some form of pathway analysis of longitudinal data. They include Attractor analysis of Boolean network of Pathway (ABP)^16^, Longitudinal Linear Combination Test (LLCT)^17^, Time-Course Gene Set Analysis (TcGSA)^18^, Gene Set Enrichment Analysis (GSEA) for time series^19^, globalANCOVA^20^, and Correlation Adjusted MEan RAnk gene set test (CAMERA)^21^. ABP allows sophisticated post-processing of sample-specific pathway scores, but it has not been implemented into an R package, which hinders the ease of its usage. LLCT is a diverse approach that builds on the principles of mixed effects modelling and can also be used with small sample sizes. However, although it can identify gene sets differentially expressed over time in association with a set of covariates, it cannot distinguish the individual covariates responsible for the difference. Earlier methods like globalANCOVA and CAMERA rely on ANOVA- and linear-modeling frameworks, respectively, but do not address potential longitudinal heterogeneity within gene sets or the correlation among repeated measurements. In contrast, TcGSA uses mixed effects modelling with maximum likelihood estimates to identify gene sets with significant variation over time and has been shown to outperform globalANCOVA and CAMERA in time-course contexts^18^. GSEA is a widely used pathway method that can take time points as phenotypes, although such continuous variables cannot be combined with categorical ones. Despite the flexibility, usability and other benefits of these methods, none of them can be used to analyze data with a variable that is unavailable for control samples (e.g. disease stage), which makes them unsuited for such study settings. They also consider pathways as simple gene sets without any further structural complexity.

Type 1 diabetes (T1D) is a complex autoimmune disease that often develops during childhood. As its progression is largely unknown, long follow-up studies involving children with the genetic risk factors are needed. Indeed, many such longitudinal studies have been carried out or are ongoing, such as Type 1 Diabetes Prediction and Prevention (DIPP)^22^, Pathogenesis of Type 1 Diabetes—Testing the Hygiene Hypothesis (Diabimmune)^23^, and The Environmental Determinants of Diabetes in the Young (TEDDY)^24^. In these studies, control samples reflect normal aging without disease development. Such studies are considerable investments of time, money, and other resources, and suitable computational tools are required to fully utilize the large-scale omics data generated in them. However, such data are not straightforward to analyze as the normal age-related development of the children causes changes that can mask the delicate biological signals related to disease progression. The appearance of certain T1D-related autoantibodies, called *seroconversion*, is the most reliable predictor of T1D^25^, but very little is known about processes leading to it. In this study, we used three publicly available longitudinal proteomic and transcriptomic datasets of children before and after seroconversion and healthy controls to demonstrate the utility of PAL in interpreting these challenging real-life datasets.

## Results

**Fig. 1:**
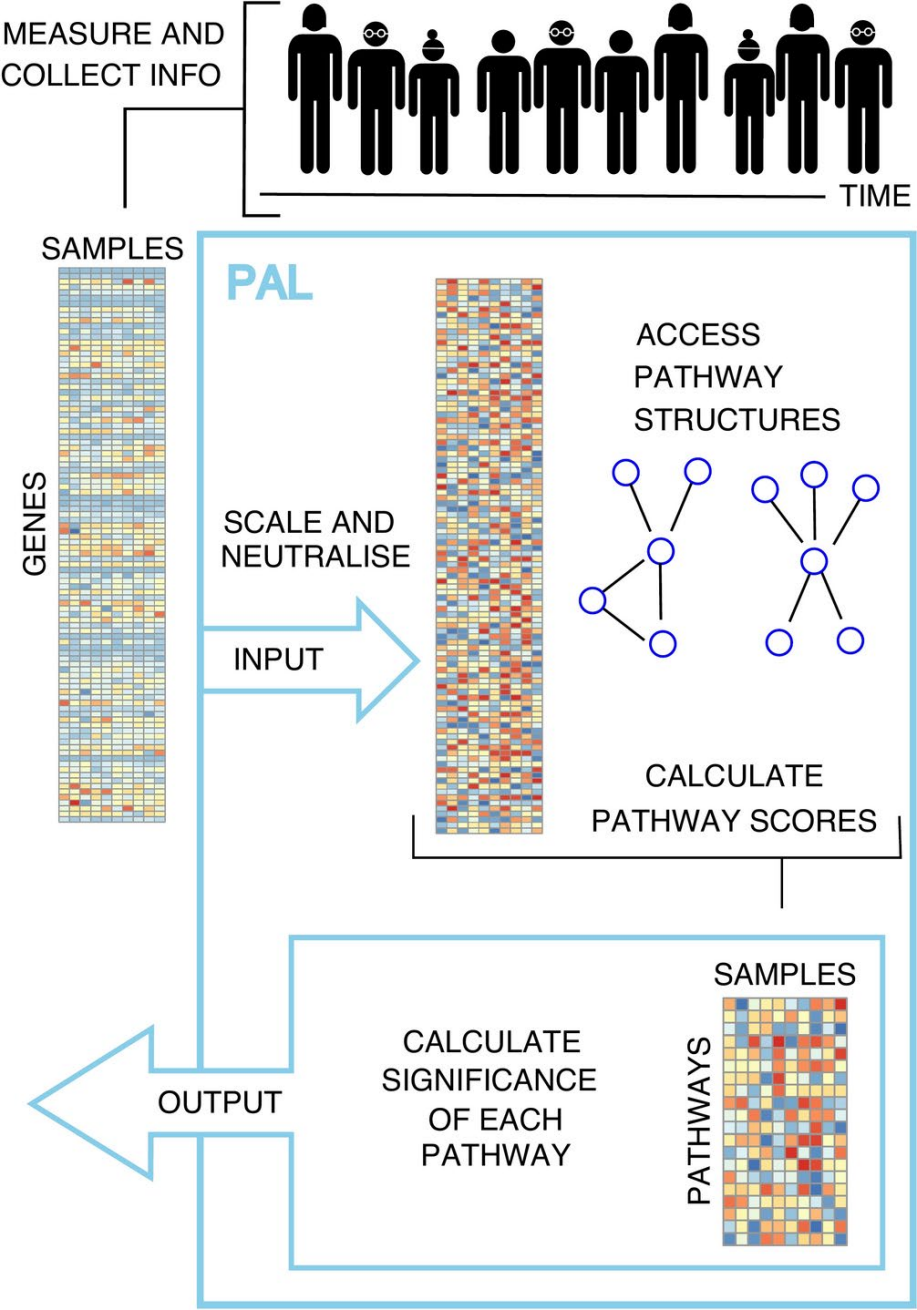
Schematic overview of the Pathway Analysis of Longitudinal data (PAL) workflow.

We have developed a new pathway analysis method PAL, which allows the analysis of complex study designs. In the PAL workflow, the effects of user-defined confounding variables are adjusted for at gene or protein level, pathway scores are calculated for all samples using pathway structures, and the significance of their association with a given main variable of interest is calculated using, by default, linear mixed effects models (Fig. 1). A more detailed description of PAL is available in the Materials and methods section.

To demonstrate the utility of PAL for identifying significant pathways with respect to different variables of interest, we analyzed simulated data and three different longitudinal datasets related to early development of T1D, involving prediabetic and healthy control children. In all these case studies, all samples were first adjusted for the normal age effect based on the healthy control samples using age as fixed effect and donor as random effect in the model fitting, and the significance for each pathway was calculated with respect to seroconversion or sample group (case vs. control), with the variable of interest as fixed effect and donor as random effect in the model fitting. Here, the control samples refer to the samples that are used as a reference to separate the effects of the studied phenomenon (i.e. disease) from other effects (e.g. normal aging). With adjusting, we refer to the removal of the effect of selected confounding factors prior to the downstream pathway analysis, so that they do not mask the delicate effect of the variable of interest.

### Performance in simulated data

**Fig. 2:**
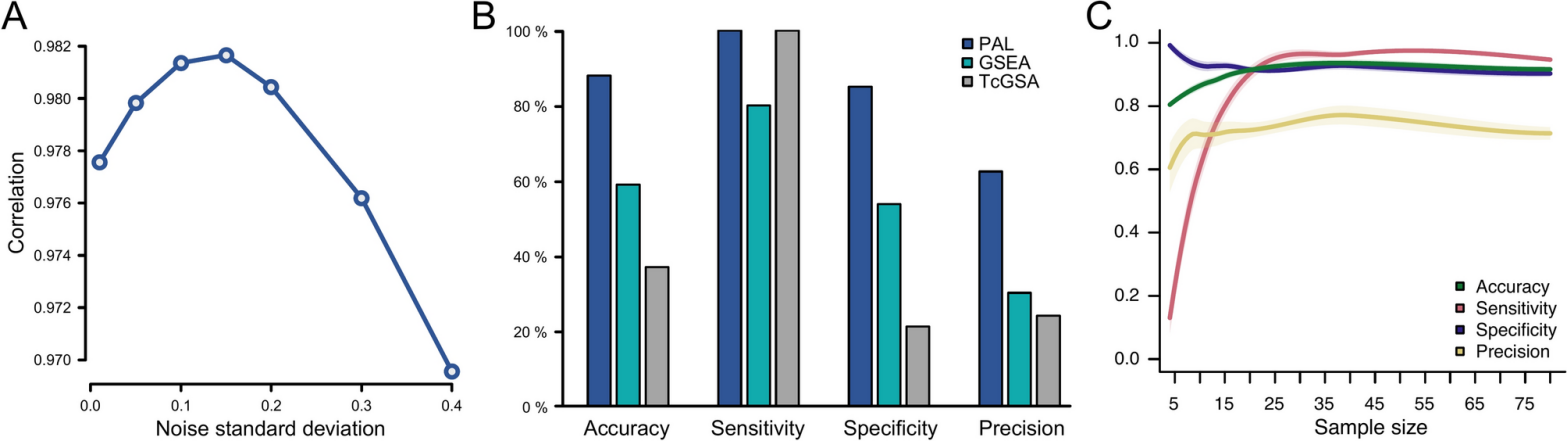
Performance in simulated data. (**A)** Pearson correlation between the known disease coefficient and the PAL-estimated disease coefficient (*y*-axis) as a function of the standard deviation of the noise level in the simulated data (*x*-axis), illustrating the impact of noise on the accuracy of PAL’s estimates. Accuracy, sensitivity, specificity, and precision of the pathway identifications at the false discovery rate of 0.05 (**B)** using PAL, GSEA, and TcGSA, and (**C)** at different sample sizes using PAL. The median and standard error of mean over ten randomly selected subsets are shown for each sample size.

To evaluate the performance of PAL in a controlled setting, we applied it to simulated data. The simulated data contained 100 pathways, among which 60 had an age effect and 20 had a disease effect, including 9 pathways with both effects. Our results show that PAL accurately estimated the coefficient of the main variable of interest (in this case, the disease effect) and the accuracy was not sensitive to noise (Fig. 2A). Furthermore, we found that the pathways with a real disease effect were ranked to the top of the list using the false discovery rate (FDR) estimates (area under the ROC curve 0.95). With 20/20 true positives and 68/80 true negatives, the accuracy, sensitivity, and specificity were all above 80% (Fig. 2B). However, we noted that the estimated FDR levels tended to be too liberal, as applying the conventional FDR cutoff of 0.05 resulted in the identification of several false positives.

We also evaluated the effect of sample size on PAL results by randomly selecting subsets of samples from the simulated data from 4 to 80. Our results show that PAL maintained its performance well with sample sizes of 20 and above (Fig. 2C). With the smaller sample sizes, the median sensitivity of PAL decreased from ~ 90% to below 20% with the smallest sample size of four, whereas the specificity, precision, and accuracy remained at comparable levels with all the sample sizes tested.

To compare PAL with other methods, we also tested TcGSA and GSEA. Although TcGSA was faster to run, we found that it had problems with controlling FDR levels (Fig. 2B); despite only 20 pathways having a disease effect, 83 out of the 100 simulated pathways had an FDR below 0.05. Accordingly, the accuracy, specificity, and precision remained below 40%. Interestingly, we noted that 14 pathways even without any age trend were identified as false positives. GSEA was not designed for complex study settings, and indeed, it identified several false positives (37/80) as well as fewer true positives (16/20). The four false negative findings were pathways with both age and disease effects, but with opposite trends (e.g. aging had a negative impact on the pathway activity, whereas disease progression induced it). The accuracy and specificity of GSEA were below 60%, with the precision being only 30%.

### Case study 1: plasma proteomics data with long follow-up time

**Fig. 3:**
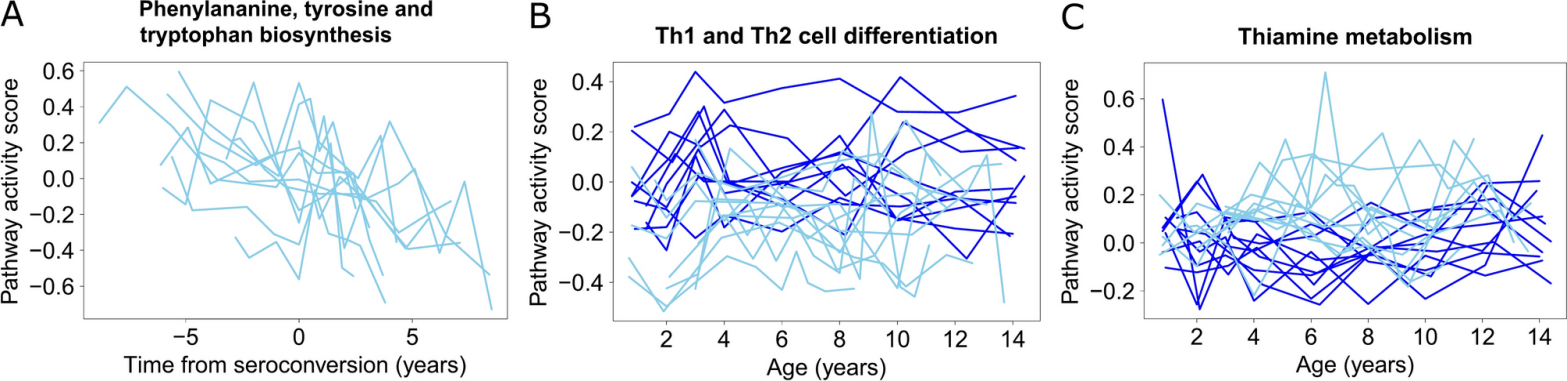
PAL results for pathways (**A**) Phenylalanine, tyrosine and tryptophan biosynthesis, (**B**) Th1 and Th2 cell differentiation, and (**C**) Thiamine metabolism. Pathway activity score estimated by PAL (y-axis) is illustrated as a function of time from seroconversion (**A**) or age (**B** and **C**) for only the case samples (**A**) or for the case and control samples (**B** and **C**). Each line represents one donor, with cases in light blue and controls in dark blue.

Application of PAL to the DAISY plasma proteomics data from 11 prediabetic and 10 healthy donors revealed nine pathways significantly associated with seroconversion after adjusting for the age effect based on the healthy control samples (FDR < 0.05, Supplementary Table 1). Among these findings, pathway ‘Phenylalanine, tyrosine and tryptophan biosynthesis’ (KEGG id hsa00400) had a particularly clear time from seroconversion effect (Fig. 3A). For comparison, TcGSA identified 77 significant pathways, of which three pathways (’Phenylalanine metabolism’, ‘Tyrosine metabolism’, and ‘PPAR signaling pathway’) overlapped with the PAL detections, whereas GSEA did not find any significant pathways (Supplementary Table 1). These results were consistent with those obtained from the simulated data, where TcGSA struggled with controlling the FDR and GSEA also suffered from false negatives.

From the comparison between the prediabetic and healthy donors, 88 significant pathways were identified with PAL (FDR < 0.05, Supplementary Table 1). Of these pathways, 75 had higher activity in the healthy control subjects (e.g. ‘Th1 and Th2 cell differentiation’, Fig. 3B) and 13 had higher activity in the prediabetic subjects (e.g. ‘Thiamine metabolism’, Fig. 3C). Out of the 88 significant detections, 25 had sample group coefficient with absolute value > 0.1. These 25 strong detections included eight disease pathways, seven pathways related to metabolism of different biomolecules, five signalling pathways, and five other pathways, namely ‘Ferroptosis’, ‘Protein export’, ‘Th1 and Th2 cell differentiation’, ‘Nucleotide excision repair’, and ‘Mineral absorption’.

As seroconversion in T1D is still not fully understood and the underlying reasons leading to it are heterogeneous and likely include both genetic and environmental factors^26^, it is difficult to determine which pathways should be detected and which ones not. Several of the nine significant pathways associated with seroconversion were related to metabolism of different biomolecules, which have been identified also in other studies. The strongest detection was ‘Phenylalanine, tyrosine and tryptophan biosynthesis’ and particularly changes related to phenylalanine and tyrosine have been found in the context of early stage T1D^26,27^ supporting also the significant pathways ‘Tyrosine metabolism’ and ‘Phenylalanine metabolism’. Similarly, the pathway ‘Bile secretion’ is supported by the literature^28^, while ‘Pancreatic secretion’ has obvious relevance. Peroxisome proliferator-activated receptors (PPARs) regulate processes like T cell differentiation and insulin secretion, supporting the significant detection of ‘PPAR signaling pathway’.

The strongest finding from the sample group comparison was ‘Alpha-Linoleic acid metabolism’, which is supported by Overgaard et al. as they showed the deregulation of diglyceride in the early stages of T1D progression^29^. The second strongest detection was ‘Ferroptosis’, referring to iron-dependent regulated cell death, which was identified only 10 years ago and is currently under intensive study; it has already been linked to the development of metabolic diseases^30^. The pathway ‘Th1 and Th2 cell differentiation’ has been strongly linked to development of T1D^31,32^. Besides ‘Alpha-Linoleic acid metabolism’, among the strong biomolecule metabolism pathway detections ‘Alanine, aspartate and glutamate metabolism’ and ‘Thiamine metabolism’ have also been linked to T1D^33,34^. In addition, different glycans and ascorbate are related to immune system, supporting the detected pathways ‘N-Glycan biosynthesis’, ‘Glycosaminoglycan biosynthesis—chondroitin sulfate / dermatan sulfate’, and ‘Ascorbate and aldarate metabolism’. Several of the strong signalling pathway detections are also related to immune system, as ‘RIG-I-like receptor signaling pathway’ works in defence against virus infections, ‘Rap1 signaling pathway’ has been shown to regulate T cell response^35^, and ‘IL-17 signaling pathway’ recruits immune cells to the site of inflammation and it has been linked to different autoimmune diseases^36^. ‘Notch signaling pathway’ has very diverse functions including regulating apoptosis and pancreatic development and its potential role in development of T1D has been discussed^37^. The eight disease-related strong pathway findings from the sample group comparison included autoimmune diseases (‘Autoimmune thyroid disease’ and ‘Type I diabetes mellitus’), rejection reaction related diseases (‘Graft-versus-host disease’, ‘Allograft rejection’, and ‘Endocrine resistance’), and infection pathways (‘Salmonella infection’ and ‘African trypanosomiasis’). While there is unlikely to be direct causality between the early stage T1D and these diseases (excluding T1D itself), they share some mechanisms or characteristics, which can explain the detection of these pathways.

Finally, we wanted to compare the PAL findings to the findings in the original study. For this, we used the detected differentially expressed proteins in the original study as an input for a standard pathway analysis method. In the original publication^38^, only two significantly differentially expressed proteins were detected at FDR of 0.05 between the prediabetic and healthy subjects. Therefore, we used a more relaxed FDR cutoff of 0.1, with which 29 differentially expressed proteins were identified. Database for Annotation, Visualization and Integrated Discovery (DAVID) analysis of them did not identify any significant KEGG pathways. Recently, Välikangas et al.^39^ identified 15 longitudinally differentially expressed proteins from the same data using their robust longitudinal differential expression method. However, again DAVID analysis of these proteins did not identify any statistically significantly enriched KEGG pathways. These results highlight the importance of pathway methods like PAL that enable discovering significant pathways from challenging datasets with complex designs and only delicate signals that remain undetected when the proteins are first analyzed individually.

### Case study 2: transcriptomics time series data from young children

The Diabimmune data^23^ containing gene expression measurements from seven prediabetic and eight healthy children were analyzed using PAL separately for the peripheral blood mononuclear cells (PBMCs), CD4 + T cells, and CD8 + T cells. Only one pathway in PBMC data, namely ‘Lysosome’, was significantly associated with seroconversion after adjusting for the age effect based on the healthy control samples (FDR < 0.05, Supplementary Table 1). For comparison, TcGSA identified 12, 44, and 68 significant pathways from the PBMCs, CD4 + T cells, and CD8 + T cells, respectively, including ‘Lysosome’ from PBMC (Supplementary Table 1). GSEA identified only one pathway with a significant association with seroconversion, which was ‘Regulation of autophagy’ in CD8 + T cells.

**Fig. 4:**
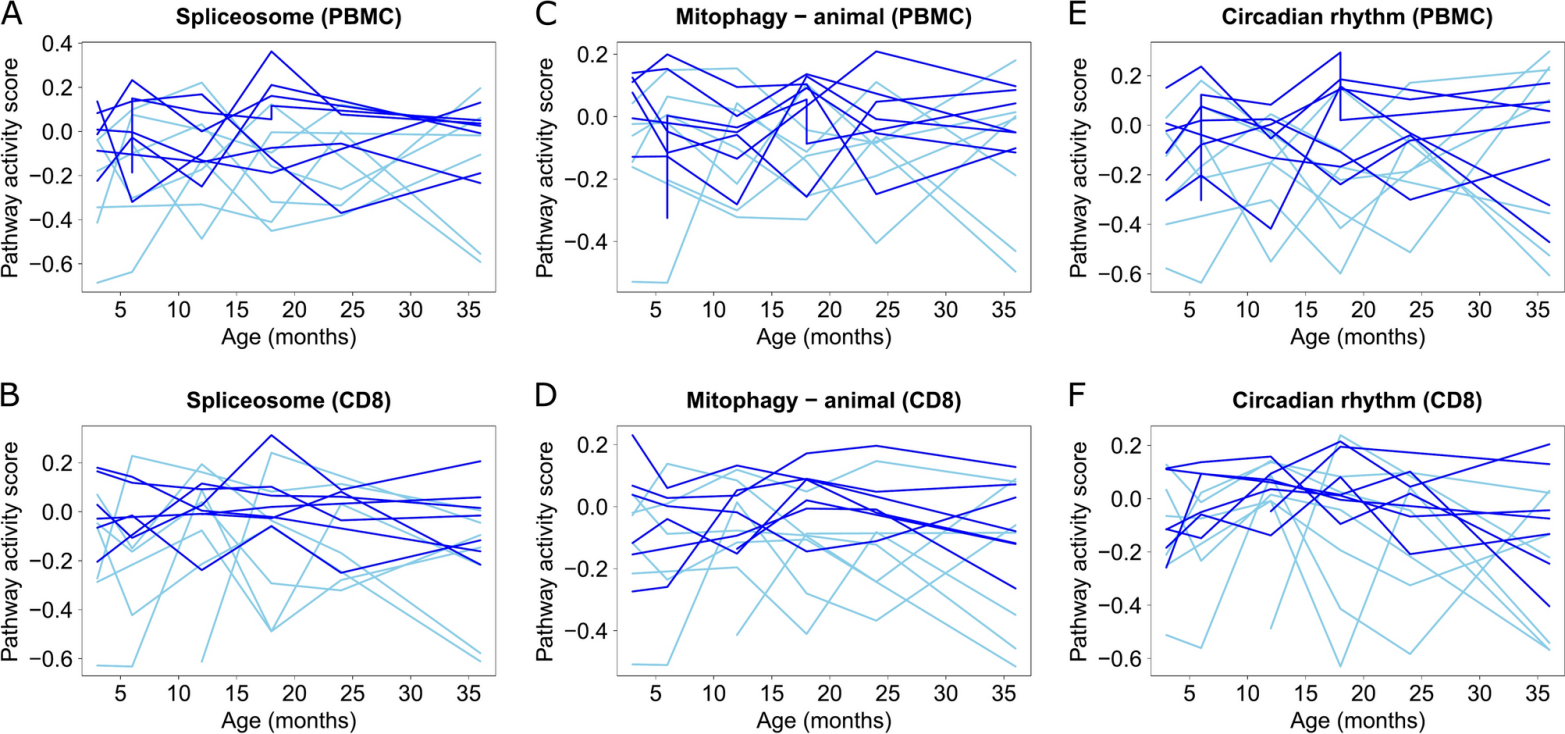
Pathway activity score (*y*-axis) as function of age (*x*-axis) of **(A–B)** Spliceosome, **(C–D)** Mitophagy—animal, and **(E–F)** Circadian rhythm in PBMC (upper panel) and in CD8 + T cells (lower panel). Case individuals are indicated with light blue lines and control individuals with dark blue lines.

In the comparison between the prediabetic and healthy control subjects, 28 pathways were significant in the PBMC samples, 17 in the CD4 + T cells, and 99 in the CD8 + T cells (Supplementary Table 1). This is in line with the original study^23^, where early changes in prediabetes already before seroconversion were reported particularly in the CD8 + T cells. Interestingly, the significant pathways from CD4 + T cells and CD8 + T cells were almost exclusively downregulated in case samples, whereas in the PBMC data 16 of the significant pathways were downregulated and 12 were upregulated in the case samples. Among the significant pathways, four had absolute sample group coefficient > 0.1 in PBMC data, three in CD4 + T cell data, and 25 in CD8 + T cell data (Supplementary Table 1). Pathways ‘Spliceosome’ (Fig. 4A–B), ‘Mitophagy—animal’ (Fig. 4C–D), and ‘Circadian rhythm’ (Fig. 4E–F) were among these strongest detections in both PBMC and CD8 + T cells, and they were all downregulated in case samples in both sample types.

The 25 strongest detections with FDR < 0.05 and the absolute sample group coefficient > 0.1 from CD8 + T cells (Supplementary Table 1) included several pathways that have been linked to the destruction of beta cells (‘Autophagy—other’, ‘Proteasome’, ‘Mitophagy—animal’, ‘JAK-STAT signaling pathway’, ‘Protein processing in endoplasmic reticulum’)^40–43^, but it is unclear if such processes are altered already at this early stage of the disease development. Three of the strong findings (‘Autophagy—other’, ‘Proteasome’, and ‘Mitophagy—animal’) are related to within-cell homeostasis and one (‘Apoptosis—multiple species’) to tissue/organ-level homeostasis. The ‘JAK-STAT signaling pathway’ is also related to apoptosis and the ‘B cell receptor signaling pathway’ to autoimmunity^44^. Changes in citrate acid cycle have been identified in children who will develop T1D in the future^45^, which supports two of the strong detections (‘2-Oxocarboxylic acid metabolism’ and ‘Citrate cycle (TCA cycle)’), as 2-Oxocarboxylic acid is part of the tricarboxylic acid (TCA) cycle. Besides the TCA cycle, ‘Oxidative phosphorylation’ is related to the ATP production. The most interesting observation among the strongest disease specific pathways was the presence of three pathways related to cardiomyopathy (‘Arrhythmogenic right ventricular cardiomyopathy’, ’Viral myocarditis’, and ’Dilated cardiomyopathy’). While increased risk of cardiomyopathy among T1D patients is strongly supported in the literature^46^, this connection with early-stage disease was surprising.

Eight of the significant detections from the PBMC data were also identified from the DAISY proteomics data. Among these eight pathways, four (‘Protein processing in endoplasmic reticulum’, ‘Autophagy—animal’, ‘Non-small cell lung cancer’, and ‘Epstein-Barr virus infection’) were downregulated in case samples of both dataset and one (‘Biosynthesis of cofactors’) was upregulated in them.

To compare the PAL findings to the findings in the original study^23^, we considered the genes reported to be differentially expressed between the sample groups across all time points (FDR < 0.05) and used the DAVID tool. With this, we did not identify any significant KEGG pathways at FDR of 0.05 from the CD8 + T cells or the CD4 + T cells, whereas 17 pathways were detected from the PBMCs (Supplementary Table 1). This is an interesting observation as PAL detected the largest number of pathways from the CD8 + T cells. One DAVID detection from the PBMC samples (‘Epstein-Barr virus infection’) overlapped with the corresponding PAL detections, and four (’Th1 and Th2 cell differentiation’, 'Th17 cell differentiation’, 'Epstein-Barr virus infection’, and 'Type I diabetes mellitus’) overlapped with the PAL detections from the CD8 + T cells.

### Case study 3: transcriptomics longitudinal data with long follow up time

Finally, we applied PAL to the BabyDiet data^47^ containing longitudinal transcriptomics PBMC data measured from 22 prediabetic and 87 healthy control children. PAL detected seven significant pathways associated with seroconversion after adjusting for the age effect based on the healthy control samples (FDR < 0.05, Supplementary Table 1), but the absolute coefficients were small indicating that the effect was relatively weak despite being significant. All of the seven significant pathways had increasing trend over time from seroconversion and four out of the seven (‘Thiamine metabolism’, ‘Lysine degradation’, ‘Purine metabolism’, and ‘Primary bile acid biosynthesis’) were related to metabolism of different biomolecules. Relatedly, the detected pathway ‘One carbon pool by folate’ supports biosynthesis and homeostasis of several amino acids^48^. For comparison, TcGSA identified 152 significant pathways, of which five overlapped with the PAL detections, whereas GSEA did not find any significant pathways (Supplementary Table 1).

In the comparison between the prediabetic and healthy control subjects, PAL identified seven significant pathways with altered activity between the groups, but again, the absolute coefficients were relatively small (Supplementary Table 1). All of the seven significant pathways were upregulated in the case samples. The strongest detection was the pathway ‘Phagosome’ while all the other significant findings were related to metabolism of different biomolecules.

Time from seroconversion was significantly associated with two pathways related to the genome and on five pathways associated with metabolism of different biomolecules. The pathway ‘One carbon pool by folate’ was the strongest among the latter. One-carbon metabolism mediated by the folate cofactor supports methionine homeostasis^48^, and methionine has been associated with early stage T1D^29^. In addition, Pácal et al. have studied connections between altered thiamine metabolism and diabetes^34^, Li et al. have provided a clear overview of roles of different amino acids, including lysine, in immune system^49^, and Ahmad and Haeusler have evaluated the roles of bile acids in glucose metabolism and insulin signalling^50^, supporting the detected pathways ‘Thiamine metabolism’, ‘Lysine degradation’, and ‘Primary bile acid biosynthesis’, respectively.

From the sample group comparison, six of the seven significant detections were related to metabolism of different biomolecules. The only exception was pathway ‘Phagosome’, which is part of the immune system. Among the detected biomolecule metabolism pathways, ‘Alanine, aspartate and glutamate metabolism’ has also been identified in another study of early T1D^33^, while ‘Retinol metabolism’ (retinol is a type of vitamin A) plays a role in immune system^51^ and two of the detections (‘Galactose metabolism’ and ‘Pentose and glucuronate interconversions’) are related to sugar metabolism. The detected ‘Biosynthesis of cofactors’ is a large pathway inducing the biosynthesis of multiple cofactors, including retinol. Interestingly, pathways ‘Alanine, aspartate and glutamate metabolism’ and ‘Biosynthesis of cofactors’ were also detected from both the DAISY and the Diabimmune PBMC datasets when the sample groups were compared. In addition, ‘Phagosome’ was detected from the DAISY and ‘Retinol metabolism’ from the Diabimmune PBMC data.

## Discussion and conclusions

In this study, we introduced a new method for pathway analysis of longitudinal data called PAL. We demonstrated the efficacy of PAL by analysing challenging longitudinal datasets related to early development of T1D with only moderate biological signals. Even from these datasets, PAL identified several relevant pathways that remained undetected in the original studies, but were well-supported by the literature. Particularly pathways related to metabolism of different biomolecules were well represented among the detections. According to our tests with simulated data, PAL outperformed other tested methods, and it was not sensitive to noise. Notably, neither TcGSA nor GSEA was designed for these types of study settings where the variable of interest is not defined for all samples, so they might perform better in other scenarios.

The tested datasets were of different origin (proteomics, RNA-sequencing, and gene expression microarrays of serum, PBMCs, CD4 + T cells, or CD8 + T cells). This, together with the heterogeneity of T1D and the very early disease stage, likely explains the modest overlaps between the detected pathways from the different datasets. When detecting pathways significantly associated with seroconversion, PAL detected the largest number of pathways in the DAISY dataset, and those detections included many processes associated with the development of T1D in the literature, suggesting the utility of proteomics for studying the early-stage development of T1D.

Our results show that PAL had reliable performance even with relatively small sample sizes, although the sensitivity and precision of the results started to decrease when the sample size was below 20. Notably, the sample size of 15 in the smallest Diabimmune dataset was on the borderline of being sufficient. Thus, those results should be interpreted with caution. Moreover, the relatively small sample size may explain why PAL only detected one pathway significantly associated with seroconversion in these data.

The benefits of breaking the model fitting into two parts (adjustment step at gene/protein level and significance step at pathway level) are three-fold. Firstly, the main variable of interest (e.g. disease stage) may not be defined for control samples, but with this approach they can still be utilized. Secondly, while the significance step has to be done at the pathway level, doing adjustment for the confounding factors already at gene/protein level supports the robustness of the approach. Finally, using only the control samples for adjustment does not mask the possibly altered effect of these variables in the case samples. In addition, PAL provides a very flexible user interface that can be used also for various goals not demonstrated here, such as multiple sample groups, and utilizes pathway structures instead of simple gene sets.

The main limitation of PAL is its inability to adjust for categorical features that are only partially shared between the sample groups. A toy example of a partially shared feature is ethnicity when cases and controls include ethnicities ‘caucasian’ and ‘hispanic’, but only cases have also some ‘asian’ samples. If both sample groups would include all three groups, there would be no problem. However, such an imbalanced study setting can also be seen as a weakness of the original study itself. Another limitation of the current version of PAL is that it relies on linear mixed effects models. While the user interface allows usage of different models (e.g. with or without random effects), by default none of them captures nonlinear trends. Linear mixed effects models provide a well-established framework for handling longitudinal dependencies and missing data, but certain biological processes may exhibit nonlinear dynamics that these models cannot capture adequately. Potential extensions include, for example, generalized additive models^52^ or spline-based mixed models^53^, which could handle more complex trends and improve the flexibility of the approach in future applications. Finally, the validation of the findings is challenging. As T1D is widely studied, but heterogeneous and not that well-understood, very many detected pathways can be linked to its development, but there is no gold standard available. However, datasets like these are the ones that most benefit from PAL analysis, which makes them realistic case studies for demonstration purposes. In addition, we used simulated data to provide more objective validation, although simulations cannot perfectly mimic the real measured data.

In conclusion, PAL is a novel pathway method suitable for analyzing datasets with complex study designs, such as longitudinal data. It is simple to use, can be applied on transcriptomics or proteomics data, uses pathway structures, detects relevant pathways even from challenging data, and allows the analysis of study designs that cannot be analyzed with other currently available pathway methods.

## Methods

This paper analyses existing, publicly available data. The accession numbers for the datasets are listed in the key resources table (Table 1). Any additional information required to reanalyze the data reported in this paper is available from the lead contact upon request.

**Table 1:** Key resources table.

Reagent or resource	Source	Identifier
DAISY longitudinal proteomic data	ProteomeXchange	PXD007884
Diabimmune longitudinal RNA-seg data	European Genome-phenome Archive	EGAC00001001443
BabyDiet longitudinal micro array data	Array Express	E-MTAB1724
PAL 1.0	This paper	https://github.com/elolab/PAL
TcGSA 0.12.10	CRAN	https://cran.r-project.org/web/packages/TcGSA/index.html
GSEA desktop application 4.3.1	Broad Institute	https://www.gsea-msigdb.org/gsea/index.jsp
DAVID 2021 update	Laboratory of Human Retrovirology and Immunoinformatics	https://david.ncifcrf.gov/
Code to generate the simulated data used in this study	This paper	https://github.com/elolab/PAL-benchmarking
Code to run the case studies with PAL	This paper	https://github.com/elolab/PAL-benchmarking
Code to run TcGSA as used in this study	This paper	https://github.com/elolab/PAL-benchmarking

### The PAL algorithm

PAL requires normalized expression data and sample group information (dummy values can be used if the study design has no sample groups) as its primary inputs; however, additional information about the samples (e.g. confounding variables) enables analysis of more complex study designs. In brief, PAL processes gene/protein expression values, incorporates structural pathway information to compute pathway-level scores, and then determines the significance of a variable of interest (e.g. disease stage) at the pathway level.

#### Gene/protein-level adjustment for confounders

The effect of confounding variables (e.g. normal aging) is adjusted separately for each gene/protein prior to the pathway analysis. By default, a linear mixed effects model is fitted to the control samples using function lmer from the R package lme4^54^. By default, user-specified confounding variables (e.g. age) are treated as fixed effects, while variables such as donor are modeled as random effects. Formally, the model is defined as **y** = **Xβ** + **Zb** + **ε**, where **y** denotes the observed expression levels of a gene/protein, **X** is the design matrix of the fixed effects, **Z** is the design matrix of the random effects, **β** is the vector of fixed effects, **b** is the vector of random effects, and **ε** is the vector of random residuals. The estimated fixed-effects coefficients **β** representing the confounding effects are multiplied by the corresponding observed values **X** and subtracted from the expression values in all samples, removing the effect of these confounders. The confounding variables can be either numeric (e.g. age or BMI), or categorical (e.g. smoking status) and many of them can be provided simultaneously. The end-user can also provide a custom model formula if the default is unsuited for their study design, and can switch to a robust linear model (function rlm from R package MASS^55^) or robust linear mixed effects model (function rlmm from R package robustlmm^56^ using the relevant arguments. It should be noted that when the number of variables in the model is high relative to the sample size, the risk of overfitting may increase.

#### Calculation of pathway scores

After the gene/protein expression values are adjusted for confounders, PAL computes the pathway scores using the the Pathway Analysis for Sample-level Information (PASI) method, which incorporates pathway structures in the analysis^10^. Within each pathway, PASI assigns a weight to each node (i.e. gene/protein) based on its processed expression value and role within the pathway topology. Nodes that are well connected within the pathway and are not present in other pathways are assigned a higher weight. For pathway activity scores (default), each relation between nodes is also assigned a value according to whether the expression levels align with their expected activating or inhibiting effects. By weighting the nodes based on pathway structure and considering the relations, PASI supports the utilization of pathway structures. Finally, the pathway scores are calculated as weighted means of these node and relation values. The output is a matrix of pathway scores with samples as columns and pathways as rows. Further details can be found in^10^. By default, PAL automatically retrieves KEGG pathways through an API.

#### Pathway-level significance testing

Finally, PAL calculates the significance of each pathway with respect to the variable of interest (e.g. disease stage) using the pathway scores. By default, a linear mixed effects model is fitted for each pathway, with the main variable of interest as a fixed effect and any random effects (e.g. donor) included as specified by the user, using the function lmer from the R package lme4. To determine a *p*-value for the main variable of interest, PAL uses a permutation-based procedure: the variable of interest is randomly permuted multiple times (n = 1000), and the coefficient obtained from the original (unpermuted) model is compared to the distribution of coefficients from the permuted models. The frequency with which the permuted coefficients exceed the original value of the coefficient is converted into a *p*-value estimate. These *p*-values are then adjusted for multiple testing using the Benjamini–Hochberg method, yielding the final false discovery rates (FDR) for each pathway. The coefficient estimates, p-values, and FDR values, together with the sample-specific pathway scores are returned for all pathways.

The PAL R package documentation in GitHub contains further instructions about the usage (https://github.com/elolab/PAL).

### Simulated data

To generate simulated data, we first created 100 pathways, each containing 5 to 60 nodes and a random set of edges, using the function sample_gnp of the R package igraph version 1.3.4^57^. We then simulated expression data for 100 case and 100 control donors, each with 2 to 8 time points. The final expression matrix with genes as rows and samples as columns can be represented as **X** = **X**_b_ + **X**_a_ + **X**_d_ + **E**, where the matrix **X**_b_ denotes the base expression, matrix **X**_a_ corresponds to the age effect (zero for genes without an age effect), matrix **X**_d_ corresponds to the disease effect (zero for genes and samples without disease effect), and matrix **E** represents noise.

To simulate the expression data, we first randomly generated a base level and standard deviation for all genes appearing in any pathway. This information was then used together with the pathways as inputs to the function generate_expression of the R package graphsim version 1.0.3^58^ to create the base expression matrix **X**_b_. We randomly selected 60 pathways to have an age effect and 20 to have a disease effect, with 9 pathways having both. The strength of the effects varied between 0.01 and 0.05 (relative to the genes’ base levels) over the affected pathways, and we added random variation over donors and genes within each affected pathway. We tested different noise levels and generated the random noise from a normal distribution with mean 0 and standard deviation of 0.01, 0.05, 0.1, 0.15, 0.2, 0.3, and 0.4. Further technical details and the specific parameters used are available in the code file *GenerateData.R* on the GitHub page containing the analysis codes used in this study (https://github.com/elolab/PAL-benchmarking).

### Longitudinal type 1 diabetes datasets

We also demonstrate the accuracy and utility of PAL with three publicly available datasets related to early development of T1D, involving longitudinal proteomics or transcriptomics data.

DAISY dataset was downloaded from ProteomeXchange^59^ with accession number PXD007884. It contains longitudinal plasma proteomics data across nine time points from 11 prediabetic and 10 healthy 0–15 year-old donors, measured using TMT-based quantitative mass spectrometry^38^. The dataset was preprocessed and log-transformed similarly as in^39^. The protein identifiers were converted to corresponding Entrez gene identifiers using R package biomaRt (version 2.48.3)^60^.

Diabimmune dataset was downloaded from European Genome-phenome Archive with accession number EGAC00001001443. It contains longitudinal transcriptomics data across five time points (3, 6, 12, 24, and 36 months of age) from 7 seroconverted and 8 healthy control children^23^. All the sample donors have increased genetic risk of T1D. Peripheral blood mononuclear cells (PBMC), CD4 + T cells, and CD8 + T cells were measured separately using RNA-sequencing. The data was normalized using the Trimmed Mean of M-values (TMM) method and log-transformed with an offset of one.

BabyDiet dataset was downloaded from Array Express with accession number MTAB1724. It contains longitudinal transcriptomics data of PBMC samples across 1 to 12 time points (median 4) from 22 seroconverted and 87 healthy control children, measured using the Affymetrix GeneChip Human Gene 1.1 ST microarray^47^. The readily available preprocessed and log-transformed data was used. While the age range of the children was wide, ranging from young babies to 10 years of age, the majority of the samples were from children under 3 years and we removed outlier samples according to age prior to the analyses. The cutoff for an outlier was defined using interquartile range (IQR) criteria, in which normal range was defined from 1st quartile—1.5*IQR to 3rd quartile + 1.5*IQR, where IQR = (3rd quartile—1st quartile). One case donor and 15 control donors had only one sample and these samples without longitudinal aspect were also removed prior to the analysis.

### Quantification and statistical analysis

The baseline pathway analysis was done by using the Database for Annotation, Visualization and Integrated Discovery (DAVID) tool (2021 update) on differentially expressed genes/proteins identified in the original studies introducing the data. Notably, no such differentially expressed genes were available from the BabyDiet study, so the baseline analysis is missing for that dataset. All analyzed genes/proteins were used as a background for DAVID. From the DAVID results, statistically significant KEGG pathways were considered. In all analyses, a finding was considered as statistically significant if it had FDR below 0.05, unless otherwise stated.

None of the alternative methods were designed for the same study settings as PAL, but TcGSA and GSEA can be used with some compromises. For TcGSA, we used the function TcGSA.LR from the R package TcGSA version 0.12.10, with the arguments subject_name, time_name, and covariates_fixed to provide donor info, disease stage, and age, respectively. The R scripts are available at https://github.com/elolab/PAL-benchmarking. GSEA cannot consider categorical and continuous features simultaneously, so donor information was completely missing from our analyses with it. Age and disease stage were provided in the input *.cls* file and the disease stage was selected as phenotype. According to the GSEA user instructions, we selected Pearson correlation as the ranking metric for longitudinal analysis. The GSEA desktop application version 4.3.1 was used. As these alternative tools throw errors if the input data includes missing values like undefined disease stage of control samples, only case samples were utilized in these tests.

## Supplementary Information

Supplementary Information.

## Data Availability

DAISY longitudinal proteomic data is available from ProteomeXchange using the identifier PXD007884, Diabimmune longitudinal RNA-seq data from European Genome-phenome Archive using identifier EGAC00001001443, and the BabyDiet longitudinal micro array data from Array Express using identifier E-MTAB1724. The tool is available from https://github.com/elolab/PAL and the scrips used in the analysis from https://github.com/elolab/PAL-benchmarking.
